# Persimmon bezoar successfully treated by oral intake of Coca-Cola: a case report

**DOI:** 10.1186/1757-1626-1-385

**Published:** 2008-12-11

**Authors:** Kazuki Hayashi, Hirotaka Ohara, Itaru Naitoh, Fumihiro Okumura, Tomoaki Andoh, Takafumi Itoh, Takahiro Nakazawa, Takashi Joh

**Affiliations:** 1 Department of Gastroenterology, Nagoya City University Graduate School of Medicine, Nagoya, Japan; 2 Department of Internal Medicine, Ohkuma Hospital, Nagoya, Japan

## Abstract

**Background:**

An 82-year-old male presented with a chief complaint of upper abdominal pain. Subsequently, a bezoar and a gastric ulcer were detected by upper gastrointestinal endoscopy.

**Case presentation:**

The bezoar was dark green in color and extremely hard, having a major axis of 7 cm. After hospitalization, 500–1000 ml/day of Coca-Cola was orally administered continuously for 3 weeks.

**Conclusion:**

Thereafter, the bezoar decreased in size to a major axis of 4 cm and showed a softening trend. Therefore, lithotripsy was thereafter carried out under endoscopy using forceps.

## Case presentation

**Figure 1:**
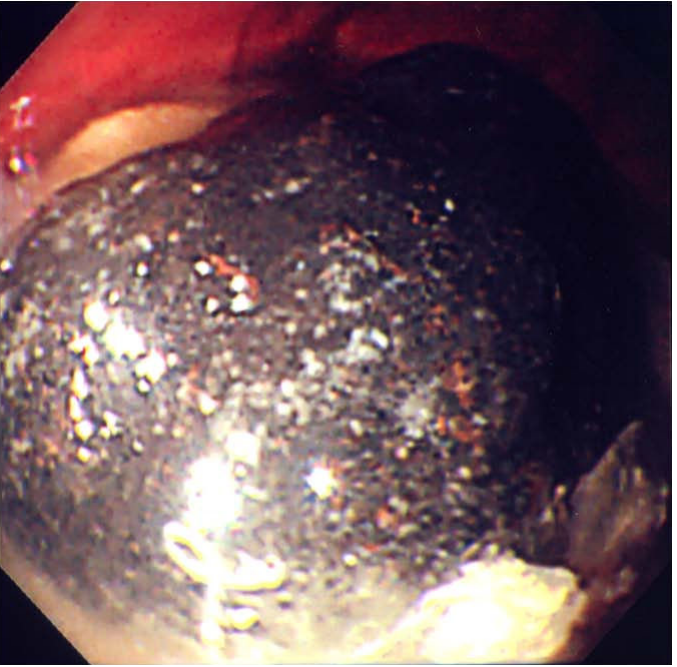
The bezoar was dark green in color and extremely hard. A large amount of residue was present in the stomach.

**Figure 2:**
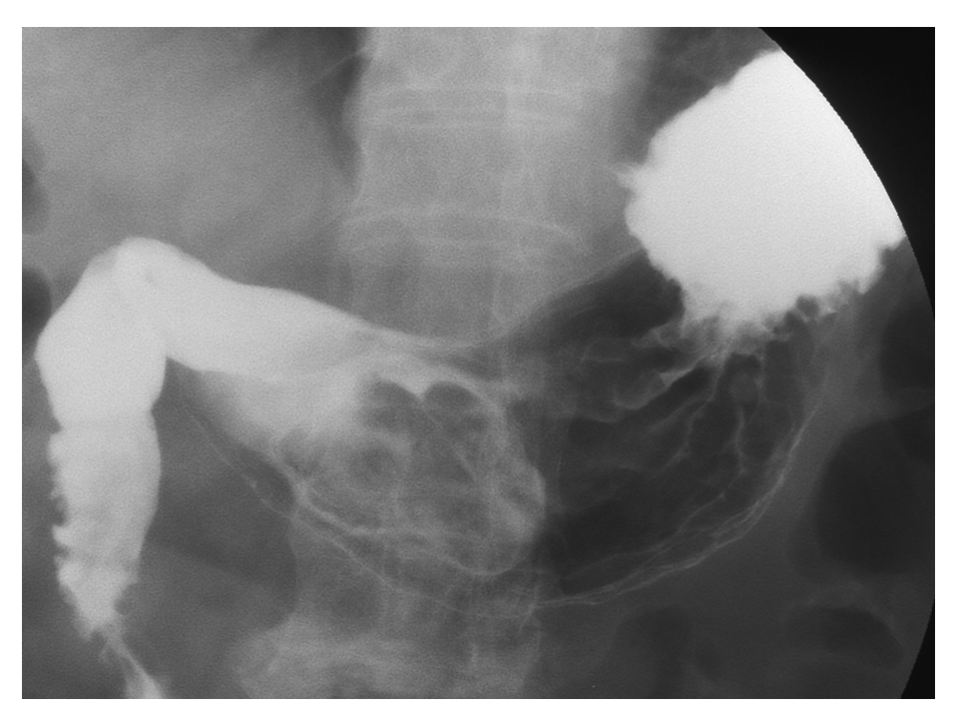
The major axis of the bezoar was found to be 7 cm by X-ray examination.

**Figure 3:**
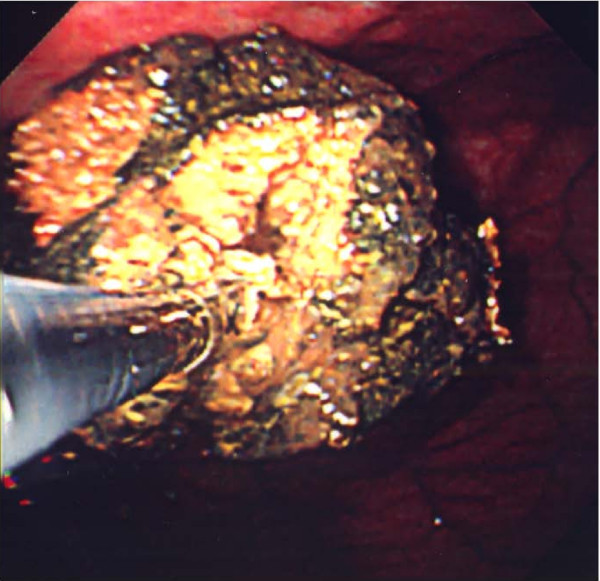
The bezoar decreased in size to a major axis of 4 cm and showed a softening trend. Therefore lithotripsy was carried out using forceps.

An 82-year-old male complained of upper abdominal pain during an outpatient visit after suffering a cerebral hemorrhage and cerebral infarction. The patient developed a cerebral hemorrhage and cerebral infarction at the age of 72, and currently demonstrated left-side paralysis. He has also been suffering from diabetes. Upper gastrointestinal endoscopy was performed after he complained of upper abdominal pain following a meal during an outpatient visit. A large amount of residue was present in the stomach, and a bezoar and a gastric ulcer were detected. The bezoar was dark green in color and too hard to scrape even with forceps(fig 1). After hospitalization and abstinence from food, a gastric X-ray examination was carried out. The major axis of the bezoar was found to be 7 cm(fig 2). After treating the gastric ulcer, a method of dissolution with Coca-Cola was scheduled. No change was observed when directly spraying the bezoar with Coca-Cola under endoscopy. An attempt was made to inject Coca-Cola into the bezoar via a puncture using a puncture needle, but the bezoar was extremely hard, which thus made puncture injection difficult. Accordingly, 500–1000 ml of Coca-Cola was continuously administered orally before each meal for 3 weeks. Endoscopy was carried out after 3 weeks of such administration. The bezoar decreased in size to a major axis of about 4 cm, the surface became uneven, and a softening trend became apparent, and therefore lithotripsy was carried out using forceps(fig 3). An analysis of the calculus was performed on the obtained bezoar. The bezoar was found to mainly consist of lecoanthrocyanis, which is one component of persimmons, thus leading to a diagnosis of a persimmon bezoar. Thereafter, the bezoar dissipated, and the patient was discharged.

## Discussion

It is believed that a bezoar develops when substances ingested as food and those such as hair that have been accidentally ingested become an insoluble calculus-like mass within the stomach. This phenomenon was first reported in 1779 by Baudamant. In Japan, the phytobezoar of a persimmon bezoar is frequently found. One type of lecoanthrocyanis, which is one component of persimmons, becomes insoluble and thereafter forms a calculus. In addition, it has been reported that bezoars are often observed in cases with diabetes and an impaired gastric excretion after gastric surgery. The present case also involved a persimmon bezoar. It appears that the stomach was likely underactive due to diabetes and unilateral paralysis, which may have led to an impaired gastric excretion.

There have been reports on the methods for treating bezoars, including surgical treatment, endoscopic lithotripsy, electrohydraulic lithotripsy, laser therapy, and even the use of extracorporeal shock wave lithotripsy (ESWL). In 2002, Ladas[1] reported a method of dissolution with Coca-Cola. The report describes that, in 5 cases of bezoars, it was found that the bezoars disappeared after irrigating approximately 3 liters of Coca-Cola within a 12-hour period via a gastric tube into the stomach. Chung[2] then reported on the injection of Coca-Cola directly into the bezoar via a puncture needle. Referring to past reports [1-9], the period from the administration of Coca-Cola until the disappearance of the bezoars was a minimum of 1 day and a maximum of 2 months. In the present case, the patient refused an indwelling gastric tube, and therefore 500–1,000 ml of Coca-Cola was administered orally. This may be the reason why it required a relatively long period for the bezoar to decrease in size.

It has not been revealed why persimmon bezoars melt after coming in contact with Coca-Cola. However, one explanation is that Coca-Cola, which contains carbonic acid and phosphoric acid, is acidic with a pH of 2.6 and thus creates a gastric environment similar to that of a normal state of gastric acid secretion[1]. Another explanation is that the fine bubbles of carbonic acid permeate the microscopically uneven surface of the bezoar[2]. Typically, a persimmon bezoar is extremely hard, which often makes it difficult to carry out an endoscopic lithotripsy. However, in the present case as well, the surface became uneven and softened, thereby allowing the successful performance of lithotripsy using forceps.

Consequently, the method of dissolving bezoars with Coca-Cola appears to be a safe, convenient, and economically feasible treatment modality.

## Consent

Written informed consent was obtained from the patient's mother for publication of this case report and accompanying images. A copy of the written consent is available for review by the Editor-in-Chief of this journal.

## Competing interests

The authors declare that they have no competing interests.

## Authors' contributions

All authors have made substantial contribution to concept this case reports.
